# Rapamycin Treatment Ameliorates Age-Related Accumulation of Toxic Metabolic Intermediates in Brains of the Ts65Dn Mouse Model of Down Syndrome and Aging

**DOI:** 10.3389/fnagi.2018.00263

**Published:** 2018-09-06

**Authors:** Nathan Duval, Guido N. Vacano, David Patterson

**Affiliations:** Department of Biological Sciences, Knoebel Institute for Healthy Aging, and Eleanor Roosevelt Institute, University of Denver, Denver, CO, United States

**Keywords:** aging, Down syndrome, Ts65Dn mouse model, metabolomics, Alzheimer’s disease

## Abstract

Down syndrome (DS), caused by trisomy of chromosome 21, is the most common genetic cause of intellectual disability. Individuals with DS exhibit changes in neurochemistry and neuroanatomy that worsen with age, neurological delay in learning and memory, and predisposition to Alzheimer’s disease. The Ts65Dn mouse is the best characterized model of DS and has many features reminiscent of DS, including developmental anomalies and age-related neurodegeneration. The mouse carries a partial triplication of mouse chromosome 16 containing roughly 100 genes syntenic to human chromosome 21 genes. We hypothesized that there would be differences in brain metabolites with trisomy and age, and that long-term treatment with rapamycin, mechanistic target of rapamycin (mTOR) inhibitor and immunosuppressant, would correct these differences. Using HPLC coupled with electrochemical detection, we identified differences in levels of metabolites involved in dopaminergic, serotonergic, and kynurenine pathways in trisomic mice that are exacerbated with age. These include homovanillic acid, norepinephrine, and kynurenine. In addition, we demonstrate that prolonged treatment with rapamycin reduces accumulation of toxic metabolites (such as 6-hydroxymelatonin and 3-hydroxykynurenine) in aged mice.

## Introduction

Down syndrome (DS) is due to partial or complete trisomy of human chromosome 21 (Hsa21) and is the most common genetic form of intellectual disability, occurring in roughly 1 in 700 live births (Parker et al., 2010; Presson et al., 2013). Individuals with DS display neurological deficits in learning and memory, are predisposed to Alzheimer’s disease (AD), autoimmune disease, blood disorders, and chronic infections, and have a lower incidence of solid tumors and hypertension (Anwar et al., 1998; Malinge et al., 2013; Bratman et al., 2014; Roberts and Izraeli, 2014; Sobey et al., 2015; Alexander et al., 2016). People with DS show many molecular and anatomical changes in the brain, including neuronal loss and the neuroanatomical hallmarks of AD, amyloid-beta plaques and neurofibrillary tangles. These changes occur most notably in hippocampus and cerebellum, and worsen with age resulting in neuroanatomical features of AD by the fourth decade of life (Lott and Head, 2001). By the time they reach 65 years of age, 68–80% of individuals with DS tested are diagnosed with dementia (Wiseman et al., 2015). Thus, people with DS represent the largest group genetically at risk for developing AD.

Many biomarkers associated with aging, such as markers for oxidative stress, accumulation of mutations, and altered DNA repair, are found in the central nervous system and periphery in individuals with DS (Lott, 2012; Patterson and Cabelof, 2012; Zigman, 2013). Marked improvements in life expectancy have been achieved, largely because individuals with DS are typically no longer institutionalized and because of improvements in, and better access to, quality medical care. However, the life expectancy of individuals with DS remains significantly reduced, and risk of mortality is higher (Coppus et al., 2008). Hematopoietic and neural stem cells taken from individuals with DS show changes characteristic of premature aging such as increased expression of pro-apoptotic genes and inflammatory genes, and reduced expression of DNA repair genes (Cairney et al., 2009). Increased accumulation of altered aspartate residues in proteins, an indicator of increased protein instability and a phenomenon associated with cellular aging, has been observed in erythrocyte membrane proteins in children with DS (Galletti et al., 2007). These findings suggest that individuals with DS experience an accelerated aging phenotype.

The Ts65Dn [Ts(17^16^)65Dn] mouse model of DS carries a partial triplication of mouse chromosome 16 (Mmu16) due to translocation of part of Mmu16 to the centromeric region of Mmu17 (Davisson et al., 1990). The Ts65Dn mouse is trisomic for approximately 100 Mmu16 genes orthologous to Hsa21 genes (Vacano et al., 2012). It is also trisomic for roughly 60 Mmu17 genes, although only ∼28 encode proteins (Duchon et al., 2011; Reinholdt et al., 2011). The Ts65Dn mouse is generally considered the best characterized DS mouse model. The mice exhibit deficits in spatial learning and memory beginning by 3 months of age. These deficits are minimized by 8 months of age but learning and memory rapidly decline as the mice age. By 12 months of age neurological deficits in learning and memory are apparent (Hyde and Crnic, 2001). Ts65Dn mice exhibit a widespread impairment of cell proliferation in cerebellum, hippocampus, skin, and bone marrow (Jablonska et al., 2006; Contestabile et al., 2009a,b). Ts65Dn mice have an increased risk of mortality and show age related declines in mobility and motor function along with other signs of premature aging reminiscent of individuals with DS (Sanders et al., 2009). The mice exhibit many features common in people with DS. For example, they have abnormal fetal brain development and reduced male fertility and exhibit many characteristics of premature aging and neuropathology reminiscent of AD (Vacano et al., 2012; Hamlett et al., 2016). The mice lose functional basal forebrain cholinergic neurons, a feature reminiscent of early onset AD (Granholm et al., 2000). Ts65Dn mice are trisomic for the amyloid precursor protein (APP) gene and although they do not develop plaques, a recent study concluded that there is an age dependent dysregulation of APP metabolism in Ts65Dn mice (Choi et al., 2009). No abnormalities could be found in APP gene expression or APP metabolite levels in brains of 4-month-old Ts65Dn mice. However, at 10- and 12-months of age, Ts65Dn mice showed elevated levels of APP and soluble APP metabolites. MRI studies reveal *in vivo* cholinergic changes possibly relevant to early onset AD in the brains of Ts65Dn mice (Chen et al., 2009). These mice also exhibit microtubule associated protein tau (Mapt, or Tau) hyperphosphorylation (Kern et al., 2011; Qian et al., 2013; Dorard et al., 2016). Recently, our laboratory conducted an analysis of the cerebellar and hippocampal proteomes in young and aged Ts65Dn mice. Our results showed expression changes in proteins involved in energy metabolism, neurotransmitter transport, and synapse function that were more dependent on age than ploidy (Vacano et al., 2018).

In recent years, rapamycin has been shown to increase the life- and healthspan of mice and to delay appearance of AD-like features in mouse models of AD (Harrison et al., 2009; Spilman et al., 2010; Miller et al., 2011; Richardson et al., 2015; Lin et al., 2017). Rapamycin is a macrocyclic immunosuppressive agent which is FDA-approved for use as an anti-rejection agent in transplant patients (Guertin and Sabatini, 2009). It forms a complex with the peptidyl-prolyl-isomerase FK506 binding protein 1A (FKBP1A) and directly inhibits signal transduction pathways involved in cell growth and proliferation by inhibition of the mechanistic target of rapamycin (mTOR) (Wullschleger et al., 2006). The mTOR signaling cascade acts as a metabolic rheostat, regulating processes required for cell proliferation (protein, lipid, and nucleotide synthesis) and suppressing catabolic activities such as autophagy (Wullschleger et al., 2006; Hartford and Ratain, 2007). Inhibition of the mTOR signaling pathways has been shown to promote longevity in *Drosophila*, yeast, and mice (Kapahi et al., 2004; Kaeberlein et al., 2005a; Harrison et al., 2009; Miller et al., 2011; Lamming et al., 2013). In particular, recent work shows that rapamycin treatment in the apolipoprotein E34 transgenic mouse model of AD rescues vascular, metabolic, and learning deficits in these mice (Lin et al., 2017). Rapamycin has proven to be an effective longevity intervention; however, there are well documented side-effects such as insulin resistance and dyslipidemias, hampering the translation of rapamycin treatment in humans (Barzilai et al., 2012; Lamming et al., 2013). In mice, these detrimental side-effects appear to diminish after prolonged exposure (>20 weeks) (Fang et al., 2013), and a number of recent publications suggest that long-term rapamycin treatment is well tolerated.

We employed high energy focused microwave (HEFM) euthanasia and high-performance liquid chromatography coupled with electrochemical detection (HPLC-EC) to detect changes in the brain metabolome of the Ts65Dn mouse model of DS, AD, and premature aging. We hypothesized that there would be changes in brain metabolites associated with trisomy and age, and that long-term treatment with rapamycin would correct these changes. Identifying novel changes temporally in these mice may provide valuable insight into the neurochemical changes associated with aging. Moreover, understanding if rapamycin alters these changes will allow us to investigate the mechanism or pathways involved in the cognitive benefits associated with rapamycin treatment. Here, we report changes in the Ts65Dn mice associated with trisomy and age consistent with neurological deficits. Among these changes, we find a disruption in the dopamine (DA) and kynurenine (Kyn) pathways previously shown to be altered due to pro-inflammatory events. These changes appear in young mice and are exacerbated in aged mice. Furthermore, rapamycin treatment appears to correct many of the metabolome changes detected in both young and old Ts65Dn mice.

## Materials and Methods

### Care and Use of the Ts65Dn Mice

All experiments were approved by the University of Denver Animal Care and Use Committee (IACUC). The care of the Ts65Dn mice and the procedures for their use follow many of the recommendations for the use of mice in gerontological research (Miller and Nadon, 2000). We chose two treatment ages, 6- and 18-months mice for metabolomics analysis. For each age group, we fed trisomic and disomic mice with either microencapsulated rapamycin or control diet (**Table 1**). Males were used exclusively for experiments because (1) Ts65Dn mice are difficult to breed; (2) Ts65Dn males are sterile and cannot be used in colony maintenance; (3) the majority of previous work using the Ts65Dn mice has been done on males. Mouse chow (Purina 5LG6) containing microencapsulated rapamycin at the dose (14 mg/kg food) previously shown to safely and effectively extend longevity, and control diet (Harrison et al., 2009; Miller et al., 2011) were purchased from Test-Diet (Richmond, IN, United States). Mice were fed control diet from weaning to 4-months of age at which point they were given rapamycin or control diet until their designated end-point.

**Table 1:** The final number of mice per rapamycin and control diet groups.

	Control		Rapamycin	
Group	6-months	18-months	6-months	18-months
Disomic	14	14	14	13
Trisomic	14	7	15	10


*The initial number of mice was 15 per group.*

Blood was collected by maxillary venipuncture monthly. A subset of blood samples from 4-, 6-, 12-, and 18-month old mice were analyzed for complete blood cell counts (CBCs) and comprehensive blood analysis as a general health check by the diagnostic company IDEXX, Inc. Additionally, five blood samples per group were sent to Dr. Martin Javors at the University of Texas Health Sciences Center San Antonio (UTHSCSA) for measurement of rapamycin levels. The mice were euthanized via HEFM irradiation directly to the head as previously described (Hunsucker et al., 2008; Vacano et al., 2018). This method euthanizes the mouse quickly and humanely, and instantaneously halts metabolic activity in the brain, preserving the *in vivo* metabolic state (Leary et al., 2013). The mice were quickly decapitated, and the brains removed; the cerebella were separated from forebrains and each placed in separate microcentrifuge tubes. We analyzed one hemisphere of the forebrain sample (excluding the cerebellum). We use “brain” from here on out to describe changes in the forebrain. Samples were flash frozen in liquid nitrogen and stored at -80°C until analysis.

### Metabolite Extraction

Metabolites were extracted from 16 mg of tissue in 1 ml of acetonitrile acidified with acetic acid (0.4%). The samples were further homogenized using sonication (Branson Sonifier Cell Disrupter 185): three 15 s intervals separated by 1-min incubation on ice at power setting no higher than 6. The homogenized samples were centrifuged at 14,000 ×*g* for 5 min at 4°C. The supernatants were collected and frozen at -80°C for 1 h, and pellets were stored at -80°C for protein content analysis (BCA colorimetric assay, Thermo Scientific) and used to normalize the metabolomic data to total protein content. The frozen samples were lyophilized in a Speedvac concentrator until all the acidified acetonitrile was removed (∼2 h). The samples were re-suspended in 200 μl of mobile phase A (see below) and centrifuged at 14,000 ×*g* for 15 min to remove insoluble material.

### High Performance Liquid Chromatography (HPLC-EC)

For the separation of brain metabolites, we employed reverse-phase HPLC using a gradient profile similar to one previously described (Kristal et al., 2007a,b). Briefly, mobile phase A is primarily aqueous: 10 g/l pentane sulfonic acid (PSA), 1% methanol (MeOH), 1 mg/l citric acid, pH 2.85. Mobile phase B is primarily organic: 50 mM lithium acetate (LiAc), 80% methanol, 10% acetonitrile, 10% isopropanol, pH 5.0. Both mobile phase solutions were filtered through 0.2 μm filters. Samples were kept at 4°C and 30 μl injected via an ESA autosampler (model 542). The samples were separated using a Tosoh Bioscience TSKgel guard cartridge and two TSKgel ODS-80Tm C-18 columns (250 mm × 4.6 mm ID, 5 μm) in series. A column and detector temperature of 30°C was maintained throughout the analysis. The analytes were detected using a CoulArray HPLC system (model 5600A, ESA) with three coulometric detector modules. Each electrochemical cell contains four flow-through coulometric detectors in series. Cell one (channels 1–4) was set to a range of potentials from 0 to 300 mV in 100 mV increments. Cells two and three (channels 5–12) were set to a range of potentials from 375 to 900 mV in 75 mV increments. The CoulArray software (ESA, version 3.10) was used for baseline correction, peak alignment, and analysis. Manual verification of peak alignment and annotation was performed to ensure correct metabolite identification. Using this system, we obtain a 2-dimensional separation of metabolites by retention time and electrochemical potential allowing resolution of co-eluting metabolites (**Supplementary Figure S1**).

### Standard Preparation and Peak Identification

Standards used for peak identification were purchased from suppliers (e.g., Sigma Aldrich) providing the highest possible purity. A metabolite database was prepared and included analyte retention time and electrochemical potential (**Supplementary Table S1**). Standards were dissolved in Milli-Q purified water (Millipore), dilute HCl or dilute NaOH, depending on solubility. Standards were run individually and in small analyte groups. This was done to determine first if the compound was detectable, and if separation from other compounds was adequate. We also “spiked” brain samples with a known amount of standard to ensure that retention times and potentials were consistent with results from standards-only runs. Peaks that did not correspond to a standard were designated “unknown” and numbered based on the order eluted off the column.

### Protein Sample Preparation

The pellets from the metabolite extracted samples were re-suspended in cell lysis buffer (10 mM Tris–HCl pH 8.3, 1 mM KCl, 2 mM EDTA, 1 mM DTT, 4% glycerol) supplemented with EDTA-free protease inhibitor [cOmplete Mini Protease Inhibitor Cocktail Tablets (Roche)]. The homogenate was centrifuged at 16,000 ×*g* for 30 min, and the supernatant was collected. The protein concentration was determined by Pierce BCA protein concentration colorimetric assay (Thermo Scientific) according to the manufacturer’s instructions and measured using a Powerwave XS2 and Gen5 software (BioTek).

### Data Processing and Analysis

Blood chemistry and general health metrics were analyzed by One-way and Two-way ANOVA when appropriate. The raw chromatographic data was baseline filtered and peak alignment was performed using CoulArray Software (ESA Bioscience) to correct for drift in peak retention times. Once the sample chromatographs were aligned, peak values (in Coulombs) were normalized to protein concentration. The normalized peak values were Pareto scaled (using the square root of the standard deviation) and centered [MetaboAnalyst 2.0 (Xia et al., 2012)]. Final normalized metabolite values were analyzed using a combination of MetaboAnalyst 2.0 software and Prism by GraphPad. Two-way ANOVA was used for statistical analysis. Data are presented as volcano plots [Log2 (Fold Change), -Log10 (*p*-value)], and box and whisker plots (mean, Q1/Q3, min/max).

## Results

### General Health Metrics

**FIGURE 1:**
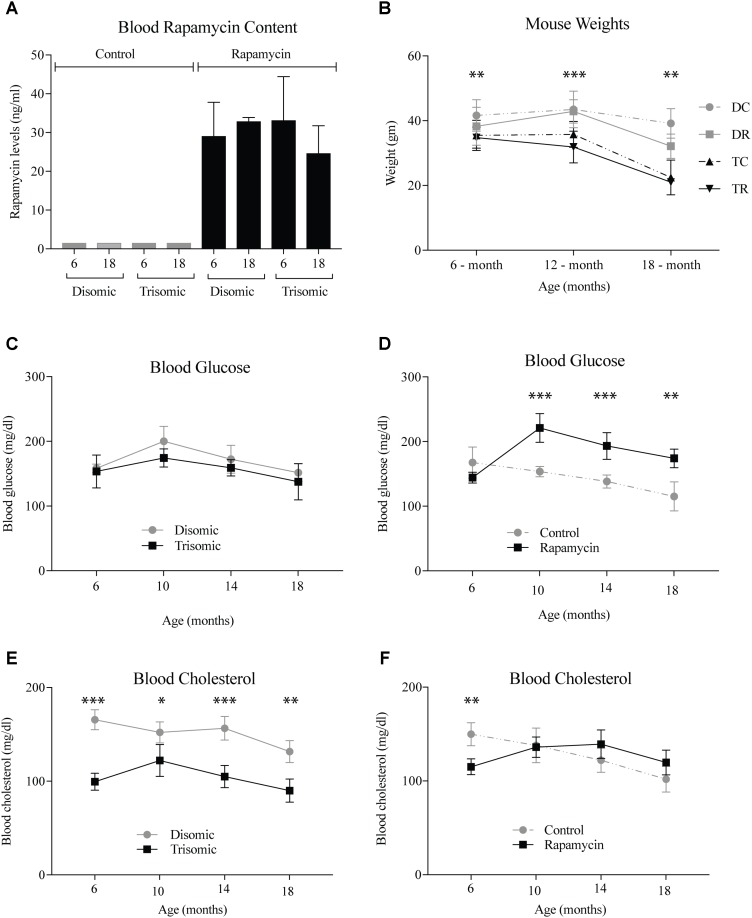
Health metrics of the Ts65Dn (trisomic and disomic) mice during rapamycin treatment study. **(A)** Rapamycin levels of the treatment groups in ng/ml. **(B)** Weights (gm) of the mice at 6-, 12-, and 18-months showing differences between disomic and trisomic mice and the two treatment groups (control/rapamycin); disomic control (DC), disomic rapamycin (DR), trisomic control (TC), and trisomic rapamycin (TR). 6-months ^∗∗^(DC–TC) and ^∗∗∗^(DC–TR); 12-months ^∗∗∗^(DC–TC), ^∗∗∗∗^(DC–TR), ^∗∗∗^(DR–TC), ^∗∗∗∗^(DR–TR); 18-months ^∗∗∗^(DC–TC) and ^∗∗∗∗^(DC–TR) are significantly different. **(C,D)** Blood glucose levels of the mice in mg/dl. **(E,F)** Blood cholesterol levels of the mice mg/dl. Data represented as mean ± SEM. Two-way ANOVA, Tukey’s *post-hoc* test **(B)**, One-way ANOVA, Tukey’s *post-hoc* test **(C–F)**, ^∗^*p* < 0.05, ^∗∗^*p* < 0.01, ^∗∗∗^*p* < 0.001, and ^∗∗∗∗^*p* < 0.0001.

A blood sample was taken the week of sacrifice by maxillary venipuncture from each mouse and a subset was sent to UTHSCSA for rapamycin analysis. As expected there was between 25 and 35 ng/ml of circulating rapamycin in the treated mice (**Figure 1A**). Mice were weighed at three intervals during the study. At 6 months, the weights of the mice were not significantly different, however, by 12- and 18-months there was a significant difference (**Figure 1B**). The Ts65Dn mice are smaller than the disomic mice, and do not lose a significant amount of weight during treatment. Interestingly, the disomic mice receiving rapamycin do have a reduction in weight at 18-months of age. Rapamycin has been proposed as a dietary intervention for weight maintenance in people who cannot diet or exercise in a safe manner (Houde et al., 2010; Moore et al., 2011).

Both complete blood count and blood chemistry was performed to assess the general health of the mice (IDEXX, Inc.). We found changes in glucose and cholesterol levels in the mice after long-term treatment with rapamycin. After 6-months of age, glucose levels rise in the rapamycin treated mice, independent of trisomy, but after 10-months, steadily decline toward pretreatment levels (**Figures 1C,D**). This increase in glucose and subsequent decrease has been witnessed in a previous study of prolonged rapamycin treatment and heart disease (Flynn et al., 2013). Cholesterol levels are significantly lower in the Ts65Dn mice compared to the disomic mice and are largely unaffected by rapamycin treatment (**Figures 1E,F**).

Since rapamycin is an immunosuppressant, we expected to see a reduction in white blood cell (WBC) levels with rapamycin treatment. Surprisingly, WBC levels were not reduced in mice treated with rapamycin relative to control diet mice, with the only exception being that 18-months disomic mice treated with rapamycin had a lower WBC count compared to 18-months control fed disomic mice (**Table 2**). We detected a decline in total WBC counts in aged (18-months) trisomic mice that is corrected in the rapamycin treated mice (**Table 2**). Analysis of these changes by two-way ANOVA showed significant reduction in total WBC counts in aged trisomic control diet mice compared to aged disomic control diet mice, which is normalized by rapamycin treatment (**Table 3**). Further analysis of WBC components (neutrophils, lymphocytes, monocytes, eosinophils, and basophils) show minimal changes in trisomic mice compared to disomic mice at 6-months, regardless of diet (**Supplementary Figure S2**). However, there were changes in WBC components in the aged trisomic mice treated with rapamycin, notably, the elevation of myeloid cells (neutrophils) and monocytes, and reduction of eosinophils. It should be noted that these values do not fall outside of normal ranges (**Supplementary Figure S2**). mTOR inhibition by rapamycin treatment may be reducing the levels of lymphocytes, thus altering the percentages of myeloid cells in the aged treated mice.

**Table 2:** White Blood Cell (WBC) counts (10^9^/l) as analyzed by IDEXX Inc., mean ± SEM.

	Control		Rapamycin	
Group	6-months	18-months	6-months	18-months
Disomic	7.16 ± 0.64	12.83 ± 0.38	8.91 ± 0.64	8.2 ± 1.19
Trisomic	9.65 ± 0.57	5.00 ± 0.33	7.54 ± 0.29	8.37 ± 2.09


**Table 3:** White Blood Cell (WBC) count two-way ANOVA *p*-values, numbers in red represent statistically significant comparisons.

		6-months			18-months			
		DR	TC	TR	DC	DR	TC	TR
6-months	DC	0.84	0.47	0.99	0.0008	0.99	0.72	0.98
	DR	–	0.99	0.95	–	0.99	0.09	0.9999
	TC	–	–	0.6748	–	–	0.02	0.97
	TR	–	–	–	–	–	–	0.99
18-months	DC	0.07	0.23	0.002	–	0.02	0.0001	0.029
	DR	–	0.95	0.99	–	–	0.30	0.99
	TC	–	–	0.53	–	–	–	0.24


*DR, disomic fed rapamycin diet; DC, disomic fed control diet; TR, trisomic fed rapamycin diet; TC, trisomic fed control diet.*

### Effects of Aneuploidy on Brain Metabolites in Young Mice: 6-Months Ts65Dn Mice and Disomic Mice

Given that roughly 100 Hsa21 ortholog genes are trisomic in the Ts65Dn mice, we expected differences in metabolite levels in Ts65Dn versus control (disomic) mice. We compared the metabolite profiles of Ts65Dn mice and disomic controls at 6-months of age (control diet) to identify significant differences in metabolites due to trisomy.

**FIGURE 2:**
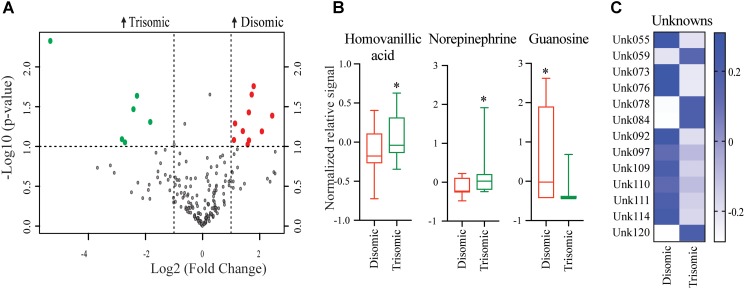
Effects of aneuploidy on brain metabolites in young mice: 6-months Ts65Dn mice and disomic mice. **(A)** Volcano plot indicating abundance and significance of metabolite changes, green dots represent metabolites elevated in trisomic compared to disomic samples, red dots represent metabolites elevated in disomic compared to trisomic samples. **(B)** Box-whisker plots (mean, Q1/3, high/low) comparing significantly changed identified metabolites. **(C)** Heatmap of significantly changed unidentified metabolites, disomic control (DC), disomic rapamycin (DR), trisomic control (TC), and trisomic rapamycin (TR). Student’s *t*-test, ^∗^*p* < 0.05.

Significant differences in abundance of some metabolites were observed (**Figure 2A**). Of the detectable 190 metabolites, we were able to identify 45. Of the identifiable metabolites, when comparing young trisomic mice to young disomic mice, we found 16 metabolites significantly changed, of these three were identified in our metabolite database. Of the metabolites we could identify, homovanillic acid (HVA) and norepinephrine (NE) are elevated and guanosine was decreased in young (6-months) trisomic mice when compared to young disomic controls (**Figure 2B**). We have not yet identified 13 of the metabolites that are significantly different in abundance due to trisomy but these may be indicative of the cognitive decline experienced by the mice as they age (**Figure 2C**).

### Effects of Aneuploidy on Brain Metabolites in Aged Mice: 18-Months Ts65Dn Mice and Disomic Mice

**FIGURE 3:**
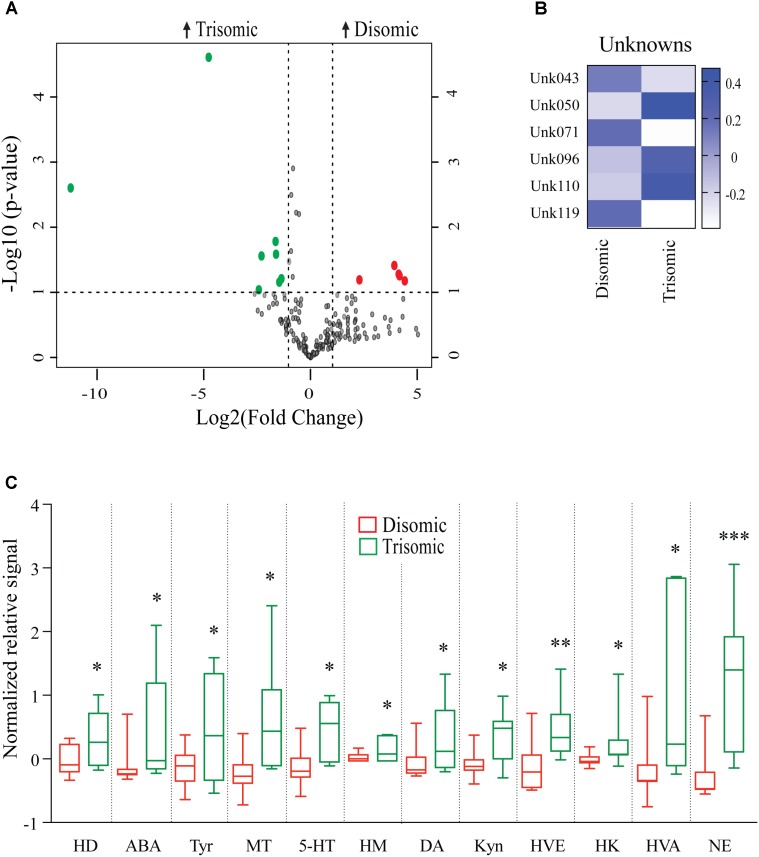
Effects of aneuploidy on brain metabolites in aged mice: 18-months Ts65Dn mice and disomic mice. **(A)** Volcano plot indicating abundance and significance of metabolite changes, green dots represent metabolites elevated in trisomic compared to disomic samples, red dots represent metabolites elevated in disomic compared to trisomic samples. **(B)** Heatmap of significantly changed unidentified metabolites in aged disomic and trisomic mice, disomic control (DC), disomic rapamycin (DR), trisomic control (TC), and trisomic rapamycin (TR). **(C)** Box-whisker plots (mean, Q1/Q2, high/low) comparing significantly changed metabolites; 6-hydroxydopamine (HD), aminobenzoic acid (ABA), tyramine (Tyr), 3-methoxytyramine (MT), 5-hydroxy-L-typthophan (HT), 6-hydroxymelatonin (HM), dopamine (DA), kynurenine (Kyn), homoveratric acid (HVE), 3-hydroxykynurenine (3HK), homovanillic acid (HVA), and norepinephrine (NE). Student’s *t*-test, ^∗^*p* < 0.05, ^∗∗^*p* < 0.01, and ^∗∗∗^*p* < 0.001.

**FIGURE 4:**
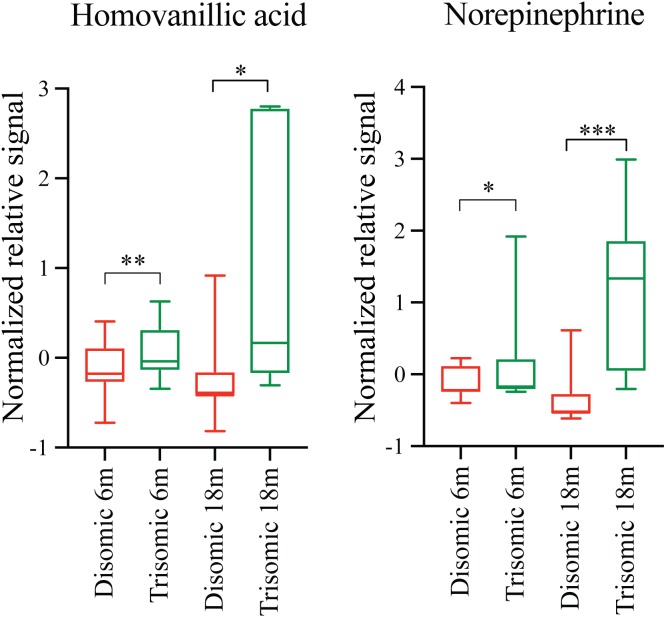
Effects of aneuploidy on homovanillic acid and norepinephrine in young and aged Ts65Dn mice and disomic mice fed control diet. Box-whisker plots (mean, Q1/Q2, high/low) showing significantly changed metabolites in both young and aged mice fed control diet; green boxes indicated trisomic, red indicated disomic. These are shown in **Figures 3** and **4**, shown here for sake of comparison (by age). Two-way ANOVA with a Tukey’s HSD *post-hoc* analysis, ^∗^*p* < 0.05, ^∗∗^*p* < 0.01, and ^∗∗∗^*p* < 0.001.

Due to the premature aging phenotype and deficits in learning and memory abilities as the mice age we expected to identify changes in brain metabolites of aged trisomic mice when compared to age-matched disomic mice. Of the 190 detectable metabolites, 18 were significantly changed (12 have been identified), most of which are elevated in trisomic brain (**Figure 3A**). In addition to the identified metabolites, we saw several currently unidentified metabolites that were changed significantly with age (**Figure 3B**). Of the identified identified metabolites that were significantly elevated in the trisomic mice, many of them represent the dopaminergic, serotonergic, and adrenergic neurotransmission systems (**Figure 3C**). Interestingly, two of the metabolites that were elevated in young trisomic brain, HVA and NE, were elevated in aged trisomic brain (**Figure 4**).

### Effects of Rapamycin Treatment in Young Mice (Ts65Dn and Disomic)

**FIGURE 5:**
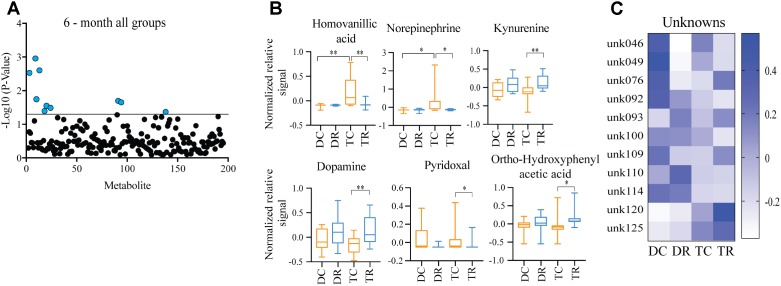
Effects of rapamycin treatment in young mice (Ts65Dn and disomic). **(A)** Two-way ANOVA of metabolite changes in young (6-months) mice comparing aneuploidy (disomic and trisomic), and treatment (control fed and rapamycin fed) groups; blue dots represent significantly changed metabolite levels, black represent unchanged metabolites. **(B)** Box-whisker plots (mean, Q1/Q2, high/low) of Tukey’s HSD *post-hoc* analysis of two-way ANOVA showing specific changes between groups. **(C)** Heatmap of significantly unidentified metabolites, disomic control (DC), disomic rapamycin (DR), trisomic control (TC), and trisomic rapamycin (TR), ^∗^*p* < 0.05 and ^∗∗^*p* < 0.01.

Comparing metabolites from 6-months control diet versus rapamycin treated mice (disomic and Ts65Dn), we found the levels of several metabolites were significantly different (**Figure 5A**). In addition to HVA and NE, we show changes in metabolites showing a response to rapamycin treatment (**Figure 5A**). Above, we showed that HVA and NE are elevated in trisomic mice when compared to disomic mice in both the young and aged condition (**Figure 4**). Here, we show that treatment with rapamycin lowers the levels of both HVA and NE to a level similar to that observed in young control diet disomic mice (**Figure 5B**). Our analysis also shows that both DA and Kyn levels are elevated in rapamycin treated 6-months groups (disomic and trisomic) versus controls (**Figure 5B**). In addition to the metabolites, we could identify, we also see a change in four currently unidentified metabolites (**Figure 5C**).

### Effects of Rapamycin Treatment in Aged Mice (Ts65Dn and Disomic Controls)

**FIGURE 6:**
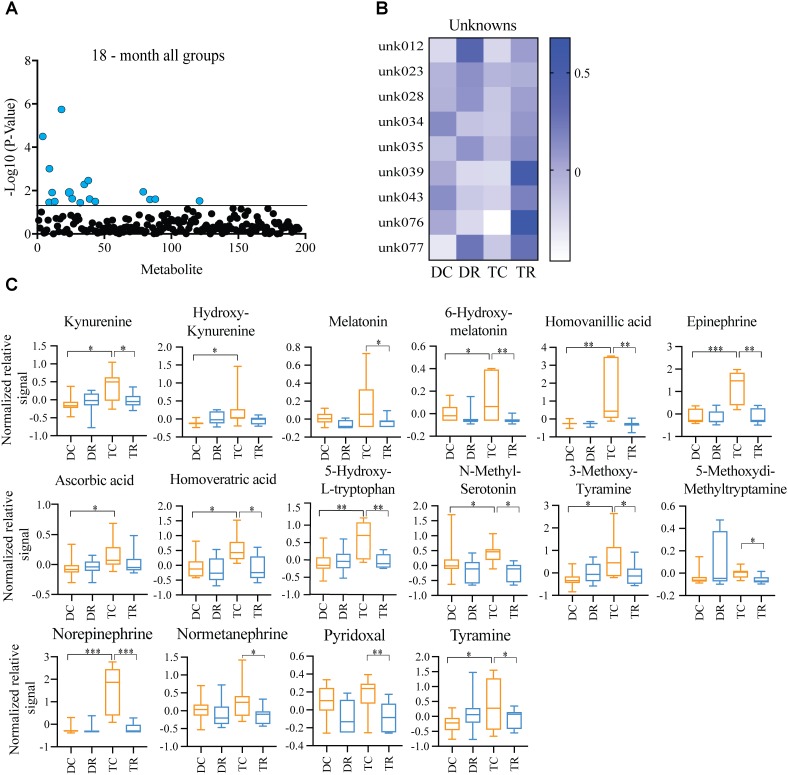
Effects of rapamycin treatment in aged mice (Ts65Dn and disomic controls). **(A)** Two-way ANOVA of metabolite changes in aged (18-months) mice comparing aneuploidy (disomic and trisomic), and treatment (control fed and rapamycin fed) groups; blue dots represent significantly changed metabolite levels, black represent unchanged metabolites. **(B)** Heatmap of significantly changed unidentified metabolites in aged disomic and trisomic mice treated with Rapamycin, disomic control (DC), disomic rapamycin (DR), trisomic control (TC), and trisomic rapamycin (TR). **(C)** Box-whisker plots (mean, Q1/Q2, high/low) of Tukey’s HSD *post-hoc* analysis of two-way ANOVA showing specific changes between groups, ^∗^*p* < 0.05, ^∗∗^*p* < 0.01, and ^∗∗∗^*p* < 0.001.

Both Ts65Dn mice and individuals with DS are susceptible to age-related neurodegeneration and premature aging. In young (6-months) mice, we found changes in metabolites, notably HVA and NE, that are due to trisomy. We also observed a reduction in the levels of these metabolites after rapamycin treatment (**Figures 4**, **5**). In aged (18-months) mice, we identified several changes in neuroactive metabolites that may indicate altered neurotransmission (**Figure 3C**). We hypothesized that we would find changes in brain metabolite levels in aged trisomic mice, and that treatment with rapamycin would act to correct levels of these metabolites. Metabolite levels in the aged (18-months) mice revealed several metabolites that are significantly different between disomic and trisomic mice, and control and treatment groups (**Figure 6A**). Nine of the metabolites were not identified (**Figure 6B**). These metabolite changes varied in levels between disomic and trisomic mice and show a differential response to rapamycin treatment (**Figure 6C**).

## Discussion

The goal of this study was to assess changes in the brain metabolome of the Ts65Dn mouse model of DS, AD, and premature aging. Combining HEFM euthanasia and HPLC-EC detection, we were able to separate and quantify 190 brain metabolites and obtain a “snapshot” of *in vivo* brain chemistry. We hypothesized that there would be metabolite changes reflecting neurological deficits due to trisomy and that these would be exacerbated in the aged mice. Additionally, we hypothesized that prolonged treatment with rapamycin, a proven longevity intervention, would ameliorate these differences. In aged trisomic mice, we observed differences in levels of metabolites representing neurotransmitter pathways involved in neurodegeneration. These include metabolites in the dopaminergic, serotonergic, adrenergic, and Kyn systems.

### General Health Metrics

The health and well-being of the mice was assessed via daily health checks, comprehensive blood chemistries and CBCs. Hyperactivation of mTOR signaling has been shown to elevate the blood glucose levels and initiate insulin intolerance by suppression of ketogenic genes in the liver and altered regulation of cell function in the pancreas (Shigeyama et al., 2008; Mori et al., 2009; Sengupta et al., 2010; Efeyan et al., 2013). Our data show an initial rise in glucose levels with a subsequent decrease after prolonged treatment, consistent with results from a study of rapamycin treatment on cardiovascular health in C57Bl/6J mice (Flynn et al., 2013). Fang et al. (2013) also showed that prolonged rapamycin treatment led to beneficial metabolic effects. Further investigation into the effect of interactions in mTORC1 and mTORC2 signaling on glucose metabolism during prolonged treatment with rapamycin is warranted.

It has been shown previously that the Ts65Dn mice develop a persistent macrocytosis and develop myeloproliferative disease (Kirsammer et al., 2008). Our data show an age-related alteration in total WBC levels in the trisomic mice versus disomic controls (**Tables 2**, **3**). In the aged mice, rapamycin treatment elevated the total WBC counts of the trisomic mice to levels observed in the disomic mice. In humans, lymphocytes are typically more sensitive to mTOR inhibition, and a decrease in lymphocytes would make the neutrophil percentage appear to be higher (Janes and Fruman, 2009). Our data suggest that this may indeed be the cause of WBC component alterations seen in the aged trisomic mice treated with rapamycin.

### Effects of Aneuploidy on Brain Metabolites

While trisomy causes many developmental deficits, including abnormal learning and memory, at 6-months of age, the Ts65Dn mice have reached their peak cognitive abilities and most resemble their disomic counterparts in some learning and memory behaviors (Hyde and Crnic, 2001). Many of the metabolites we detect show a significant difference between disomic and trisomic mice at this age but have not yet been identified. Of those that we have identified, HVA and NE are elevated and guanosine is decreased in trisomic mice. HVA is a major catecholamine metabolite intimately associated with DA metabolism. Altered HVA metabolism has been reported in DS, AD and other neurodegenerative disorders such as Parkinson’s disease (PD) and Huntington’s disease (HD) (Kay et al., 1987; Schapiro et al., 1987; Stahl et al., 1987; García Ruiz et al., 1995; Vermeiren et al., 2014; Morimoto et al., 2017; Stefani et al., 2017). Measurement of these compounds in cerebrospinal fluid (CSF) or post-mortem brain tissue typically shows a reduction in HVA levels, corresponding with reduced levels of DA. Elevated levels of HVA have been associated with dysfunction in the DA transporter (DAT) resulting in slower DA reuptake and increased oxidative stress. Interestingly, HVA is elevated (along with other catecholamine metabolites) in urine of neuroblastoma patients (Lunardi et al., 2009; Barco et al., 2014; Stefani et al., 2017).

We also observed elevated levels of NE, a catecholamine metabolite synthesized from DA and critical in the noradrenergic system. NE is produced primarily by cells in the locus coeruleus (LC), a region of the brain greatly affected in AD, PD, and DS (Marcyniuk et al., 1988; German et al., 1992; Granholm et al., 2010; Lockrow et al., 2011). Loss of neurons in the LC corresponds to increased deposition of amyloid beta (Aβ) in a mouse model of AD in a NE dependent manner (Heneka et al., 2010). Our reported elevation in NE may indicate a disruption in the DA neurotransmission system. It has previously been shown that impairment of mTORC2 signaling increases prefrontal cortex concentrations of NE (Siuta et al., 2010). Although rapamycin is considered a specific mTORC1 inhibitor, inhibition of mTORC2 due to long-term *in vitro* rapamycin treatment has been observed (Sarbassov et al., 2005). However, Halloran et al. (2012) showed memory enhancement and no impairment in mTORC2 activity nor disruption in NE inputs from mTOR inhibition after long-term rapamycin treatment. Monoamine neurotransmitters (NE specifically) have been shown to be elevated in DS and indicate disrupted monoamine turnover and changes to functional brain activity (Kay et al., 1987; Schapiro et al., 1987). Taken together, our data show an early disruption in the metabolism of DA metabolites, specifically HVA and NE, in young trisomic mice.

Previous work comparing metabolites extracted from brains of patients with DS and AD showed reductions in NE, HVA, DA, and other dopaminergic metabolites (Reynolds and Godridge, 1985; Godridge et al., 1987). Our data show elevations in NE and HVA and suggest a widespread neuronal dysregulation affecting these neurotransmitter systems that precedes the neuronal loss associated with AD and subsequent reductions in these metabolites levels. It may be that the post-mortem interval between harvest and analysis may have allowed for degradation of the metabolites in the above studies on human tissues. Our study employed HEFM which halts all metabolic activity in the brain reducing post-mortem changes in metabolites (Hunsucker et al., 2008).

### Effects of Aneuploidy on Brain Metabolites in Aged Mice

One goal of this study was to understand the metabolome changes in aged Ts65Dn mice. We identify many metabolites that were changed, several of which are key metabolites in the dopaminergic, serotonergic, and Kyn systems. Metabolites of the dopaminergic system include precursors and degradation products of DA and are critical to DA-related neurotransmitter activity. These metabolites include DA, 6-hydroxydopamine (6HD), HVA, NE, 3-methoxytyramine (MT), and tyramine (Tyr). Our data show that several of these metabolites are elevated in 18-months Ts65Dn trisomic mice when compared to their disomic counterparts. Metabolites associated with the serotonergic system changed in aged trisomic mice: these include 5-hydroxy-L-tryptophan (HT), 6-hydroxymelatonin (HM), and melatonin (Mel). Additionally, we find metabolites associated with the Kyn pathway, including Kyn metabolite 3-hydroxykynurenine (HK). Of particular interest are the elevated levels of known neurotoxic intermediates such as 6HD, an amine derivative toxic to dopaminergic neurons and HK a neurotoxic metabolite of Kyn metabolism. Hydroxydopamine is often used to induce a PD phenotype in mouse models by causing dopaminergic neuronal death. Neuronal death from endothelial inflammation due to pro-inflammatory cytokines [such as interleukin-1β (IL-1β), interleukin-6, and tumor necrosis factor-α (TNFα)] (Fu et al., 2017) may be responsible for the elevated levels of neurotoxic 6HD and HK observed in the aged trisomic mice. 3-hydroxykynurenine is a metabolite of the Kyn pathway and is a major metabolite in the degradation of tryptophan. Elevated levels of HK have been associated with excitotoxicity, and HK is a known generator of highly reactive radicals such as quinolinate (Guidetti and Schwarcz, 1999). Patients with AD have elevated levels of HK, and blocking HK formation using a small molecule prodrug inhibitor of kynurenine 3-monooxygenase (KMO) reduces the neurodegenerative events in transgenic mouse models of AD (Zwilling et al., 2011; Amaral et al., 2013; Schwarz et al., 2013). Activated microglia predominantly express KMO which catalyzes the conversion of Kyn to HK in response to inflammatory signals [TNFα and interferon-gamma (IFNγ)] and further activation of indoleamine 2,3-dioxygenase (IDO) by IFNγ may drive the production of reactive radicals by triggering tryptophan degradation (Guillemin et al., 2005; Connor et al., 2008; Parrott and O’Connor, 2015). Interestingly, genes for four of the six interferon receptors are triplicated in DS and elevated interferon signaling has been reported in individuals with DS (Reboul et al., 1999; Tanaka et al., 2012). Maroun (1980, 1995) employed the Trisomy 16 (Ts16) mouse model of DS to test the hypothesis that elevated interferon signaling is the probable antagonist in the deficiencies experienced by individuals with DS. Their efforts to regulate interferon activity by gene knockout resulted in improved development and survival of cortical neurons in the Ts16 mouse model (Maroun et al., 2000). Recently, using complementary transcriptome analysis and shRNA loss-of-function screens, Sullivan et al. (2016) have reinvigorated the idea of aberrant IFN signaling in DS as a potential mechanism for the comorbidities found in DS. In a similar vein, we believe that disruption in Kyn metabolism may be a marker for chronic pro-inflammatory (elevated TNFα, IL-1β, and IFNγ) signaling caused by the triplication of IFN receptors in individuals with DS and may provide a therapeutic target.

A recent study employed an electrochemical (amperometric) detection method coupled with HPLC to analyze monoamine metabolites in the dopaminergic, serotonergic, and (nor)adrenergic neurotransmission systems in brains from a mouse model of DS (Dekker et al., 2017). They report changes mainly associated with aging, rather than aneuploidy, consistent with the changes we observed in our analysis. Another study using HPLC-EC to compare CSF samples from patients with AD, or mild cognitive impairment (MCI) subjects to control subjects showed several changes in metabolites involved in tryptophan, tyrosine, and purine metabolism (Kaddurah-Daouk et al., 2013). The pathways affected are consistent with those affected in the Ts65Dn mice. Tryptophan, tyrosine and purine metabolism are integrated with Kyn, serotonin, and DA metabolism.

### Effects of Rapamycin Treatment

Rapamycin treatment has been shown to extend lifespan and improve health in evolutionarily divergent species, including some models of neurodegeneration (Kaeberlein et al., 2005b; Bjedov et al., 2010; Miller et al., 2011; Wilkinson et al., 2012), and it has a positive effect on cognition in aging models and has shown promise as an intervention to improve health and cognition (Caccamo et al., 2010; Spilman et al., 2010; Halloran et al., 2012; Majumder et al., 2012). In addition to the benefits of rapamycin, potentially via immunomodulation, there are side-effects associated with short-term treatment, which diminish after prolonged treatment (Fang et al., 2013; Flynn et al., 2013). Our results show rapamycin treatment in young mice elevates the levels of Kyn, DA, and ortho-hydroxyphenylacetic acid (DA derivative), and lowers pyridoxal, the biologically active form, pyridoxal phosphate, is required for Kyn metabolism (Majewski et al., 2016), in both disomic and trisomic mice, potentially due to the normalization of upstream inflammatory responses.

In aged trisomic mice, we detected elevated levels of HVA, NE, and other deleterious metabolites (6HD and HK), and these may be indicative of dysregulated inflammation and elevated levels of oxidative radicals in the brain. Long-term rapamycin treatment in aged trisomic mice corrected the level of these metabolites, which may reduce chronic inflammation. Long-term rapamycin treatment in 18-months old C57BL6/129svJ mice was shown to reduce age-related cognitive deficits by reducing the pro-inflammatory cytokine IL-1β and enhancing *N*-methyl-D-aspartate (NMDA) signaling (Majumder et al., 2012). Furthermore, rapamycin treatment has been shown to be protective against PD associated neuronal cell loss by reducing L-DOPA associated dyskinesia by enhancing gamma-aminobutyric acid (GABA) signaling in the basal ganglia (Santini et al., 2009; Malagelada et al., 2010).

The DA and Kyn systems play an important role in neurotoxicity and neurodegeneration. Reductions in DA and the dopaminergic system have been implicated in neurological disorders such as PD, HD, and multiple sclerosis (MS) and may play a critical role in aging-related neurodegeneration (Yates et al., 1983; LeWitt et al., 1992; Havelund et al., 2017). Rapamycin has been shown to elevate midbrain DA (and other monoamine) levels after prolonged treatment in C57Bl/6 mice (Halloran et al., 2012). Alterations in Kyn metabolism that have been implicated in AD, HD, and PD may play a role in other psychiatric disorders, such as depression and schizophrenia (Beal et al., 1992; Guillemin et al., 2003; Nemeth et al., 2006; Myint et al., 2007; Schwarz et al., 2013). The Kyn pathway may act as a rheostat for inflammation and immune response and may have both systemic and CNS implications in aging (Amaral et al., 2013; van der Goot and Nollen, 2013; Capuron et al., 2014; Cervenka et al., 2017).

Due to a lack of metabolomics studies in young individuals with DS, or longitudinal studies as individuals age, it remains to be determined how well our results recapitulate the DS metabolome. However, we identified metabolic pathways that may serve as biosensors for neurotoxic events, perhaps induced by elevated neuroinflammation and oxidative stress. In addition to metabolic changes associated with aging, our results show that long-term rapamycin treatment corrects the levels of these metabolites. Understanding the connection, if any, with recent studies indicating interferon signaling as a major causative factor in DS related pathologies will be important (Sullivan et al., 2016). Interferons may be causing chronic inflammation in DS by disrupting the brain metabolome. Interferons have been shown to modulate Kyn metabolism through KMO and IDO, causing inflammation induced catabolism of tryptophan and accumulation of neurotoxic Kyn intermediates. Rapamycin, an immunosuppressant, would likely reduce interferon induced inflammation caused by triplication of interferon receptors in DS. An analysis of interferon activity in the Ts65Dn and DS brain may provide insights into inflammation and the role of the mTOR pathway in inflammatory events in the aging brain. Our results may be informative for developing new therapies, and for further investigation of rapamycin and rapalogs.

## Author Contributions

ND, GV, and DP—substantial contributions to the intellectual content, design and acquisition of the work; interpretation and analysis of work; and drafting, editing, and final approval of the manuscript.

## Conflict of Interest Statement

The authors declare that the research was conducted in the absence of any commercial or financial relationships that could be construed as a potential conflict of interest.
