# Supplementary material for: Rapamycin Treatment Ameliorates Age-Related Accumulation of Toxic Metabolic Intermediates in Brains of the Ts65Dn Mouse Model of Down Syndrome and Aging
Source: Front Aging Neurosci. 2018 Sep 6;10:263. doi: 10.3389/fnagi.2018.00263 (PMC6135881; doi:10.3389/fnagi.2018.00263)
Supplement: Supplementary file 1 [file Data_Sheet_1.DOCX]

Supplementary Material

Rapamycin Treatment Ameliorates Age-Related Accumulation of Toxic Metabolic Intermediates in Brains of the Ts65Dn Mouse Model of Down syndrome and Aging

Nathan Duval^1^, Guido N. Vacano, and David Patterson

*** Correspondence:** Corresponding Author: email@uni.edu

## Supplementary Figure


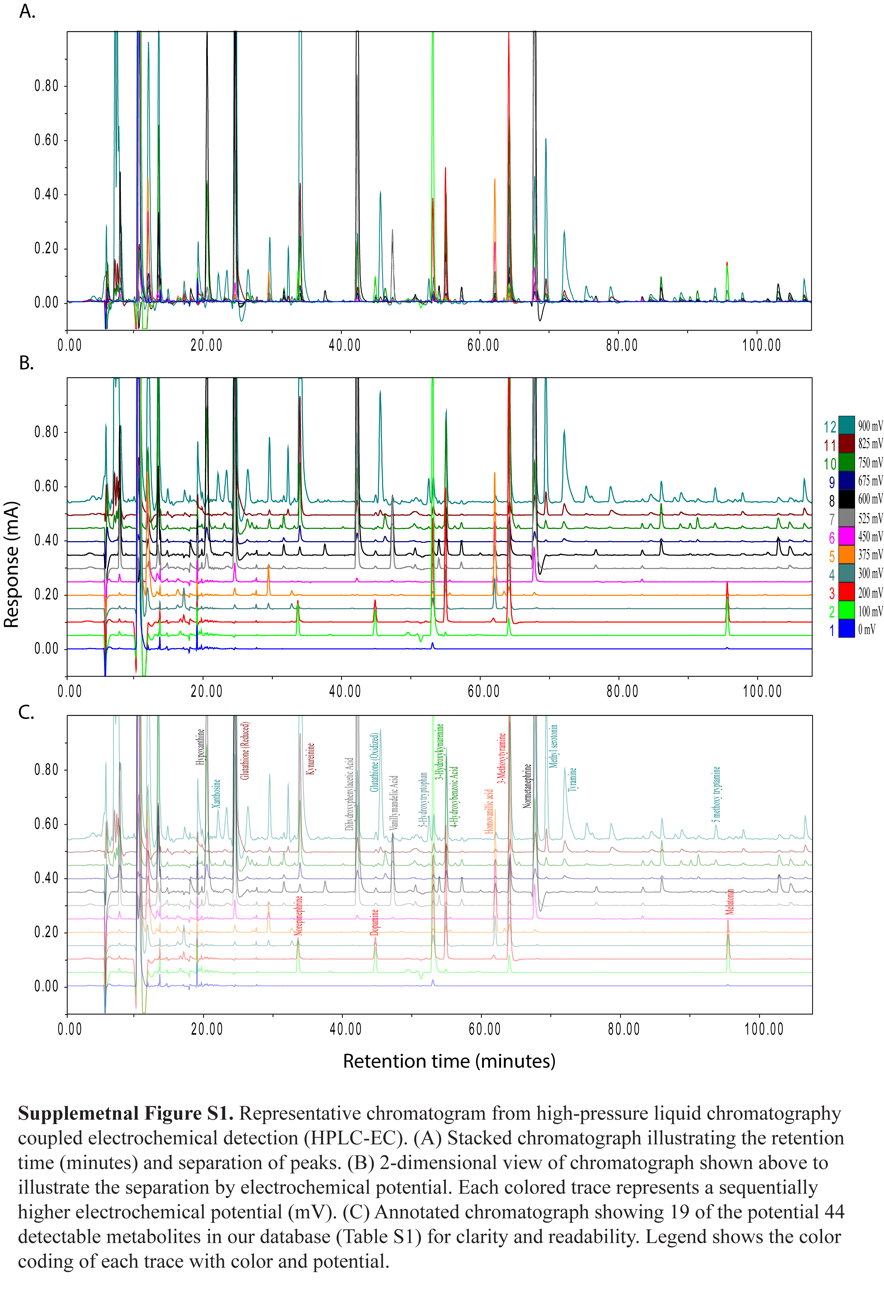


##
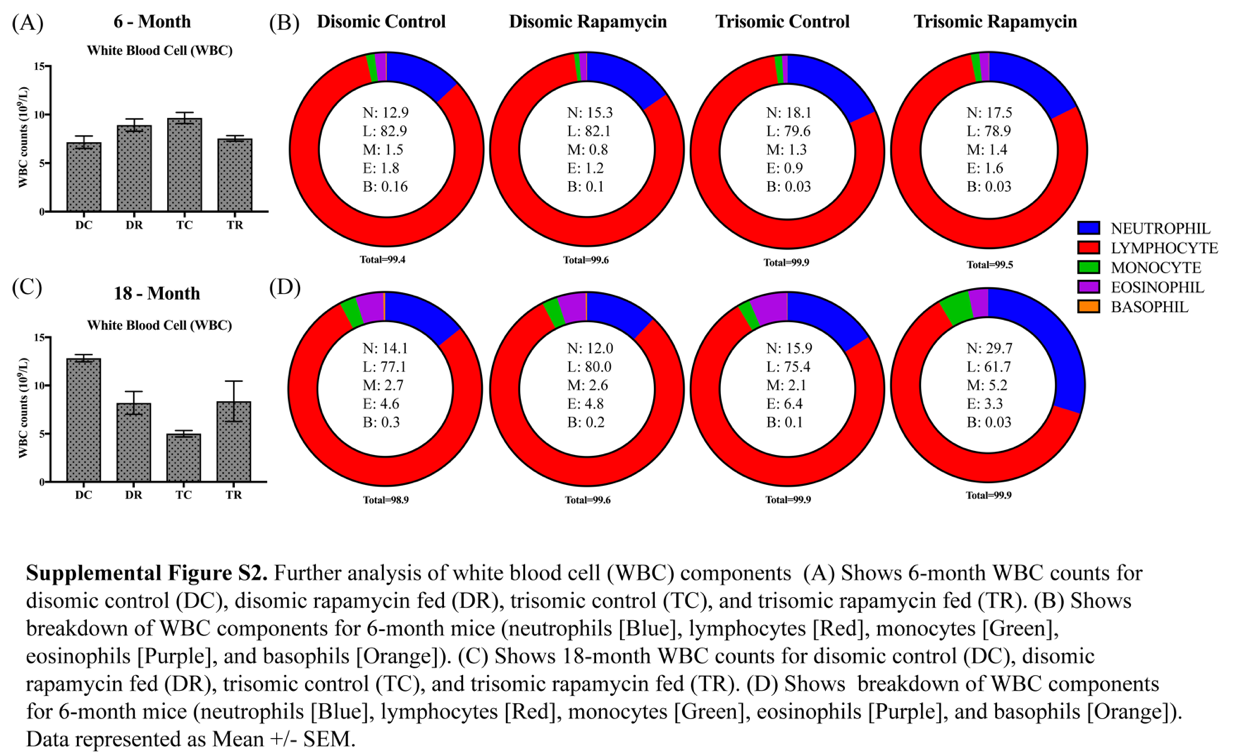


## Supplementary Table

| *Table 1S. Library of metabolites detectable by HPLC-EC.* | | | | | |
| --- | --- | --- | --- | --- | --- |
| Metabolite | **HMDB** | **PubChem** | **KEGG** | **Retention time (minutes)** | **Electrochemical potential (mA)** |
| Uric acid | HMDB00289 | 1175 | C00366 | 17.0 | 4 |
| Cysteine | METPA0075 | NA | C00736 | 18.4 | 12 |
| Ascorbic acid | HMDB00044 | 54670067 | C00072 | 19.1 | 3 |
| Xanthine | HMDB00292 | 1188 | C00385 | 20.3 | 9 |
| Hypoxanthine | HMDB00157 | 790 | C00262 | 22.2 | 12 |
| Glutathione | HMDB00125 | 124886 | C00051 | 23.4 | 11 |
| Xanthosine | HMDB00299 | 64959 | C01762 | 23.6 | 9 |
| Vanillic acid | HMDB00484 | 8468 | C06672 | 24.1 | 9 |
| Homogentisic acid | HMDB00130 | 780 | C00544 | 32.6 | 2 |
| Guanosine | HMDB00133 | 6802 | C00387 | 34.6 | 11 |
| Norepinephrine | HMDB00216 | 439260 | C00547 | 34.7 | 3 |
| L-Kynurenine | HMDB00684 | 161166 | C00328 | 36.7 | 11 |
| Guanine | HMDB00132 | 764 | C00242 | 38.5 | 9 |
| Dihydroxyphenylacetic acid | HMDB00755 | 9378 | C03672 | 42.8 | 9 |
| 2-Aminobenzoic acid | HMDB01123 | 227 | C00108 | 43.1 | 5 |
| Epinephrine | HMDB00068 | 5816 | C00788 | 43.2 | 3 |
| Dopamine | HMDB00073 | 681 | C03758 | 44.6 | 3 |
| Oxidized glutathione | HMDB03337 | 975 | C00127 | 45.8 | 12 |
| 7-Methylguanine | HMDB00897 | 11361 | C02242 | 46.3 | 9 |
| Vanillylmandelic acid | HMDB00291 | 736172 | C05584 | 46.3 | 7 |
| Pyridoxal | HMDB01545 | 1050 | C00250 | 47.7 | 11 |
| 3,4-Dihydroxybenzeneacetic acid | HMDB01336 | 547 | C01161 | 48.2 | 4 |
| Ortho-Hydroxyphenylacetic acid | HMDB00669 | 11970 | C05852 | 48.7 | 11 |
| 3-Hydroxyanthranilic acid | HMDB01476 | 86 | C00632 | 50.3 | 3 |
| 5-Hydroxy-L-tryptophan | HMDB00472 | 144 | C01017 | 53.3 | 12 |
| 3,4-Dimethoxyphenylethylamine | HMDB41806 | 8421 | N/A | 54.6 | 6 |
| 3-Hydroxykynurenine | HMDB00732 | 89 | C02794 | 54.8 | 2 |
| 4-Hydroxybenzoic acid | HMDB00500 | 135 | C00156 | 57.2 | 10 |
| 5-Methoxydimethyltryptamine | HMDB02004 | 1832 | C08309 | 57.4 | 11 |
| N-Acetylserotonin | HMDB01238 | 903 | C00978 | 59.3 | 3 |
| 6-Hydroxydopamine | HMDB01537 | 4624 | N/A | 60.5 | 5 |
| Homovanillic acid | HMDB00118 | 1738 | C05582 | 62.6 | 4 |
| 3-Methoxytyramine | HMDB00022 | 1669 | C05587 | 65.1 | 3 |
| p-Hydroxyphenylacetic acid | HMDB00020 | 127 | C00642 | 66.8 | 9 |
| Normetanephrine | HMDB00819 | 1237 | C05589 | 67.8 | 8 |
| Metanephrine | HMDB04063 | 21100 | C05588 | 68.5 | 9 |
| 4-Hydroxy-3-methoxybenzenemethanol | HMDB32012 | 62348 | C06317 | 68.9 | 9 |
| N-Methylserotonin | HMDB04369 | 150885 | C06212 | 70.6 | 12 |
| Tyramine | HMDB00306 | 5610 | C00483 | 71.5 | 12 |
| 3-Indolepropionic acid | HMDB02302 | 3744 | N/A | 71.6 | 9 |
| 6-Hydroxymelatonin | HMDB04081 | 1864 | C05643 | 78.6 | 11 |
| Homoveratic acid | HMDB00434 | 7139 | N/A | 83.5 | 7 |
| Tryptophanol | HMDB03447 | 10685 | C00955 | 85.8 | 9 |
| 5-Methoxytryptophan | HMDB02339 | 151018 | N/A | 93.1 | 12 |
| Melatonin | HMDB01389 | 896 | C01598 | 93.3 | 3 |
